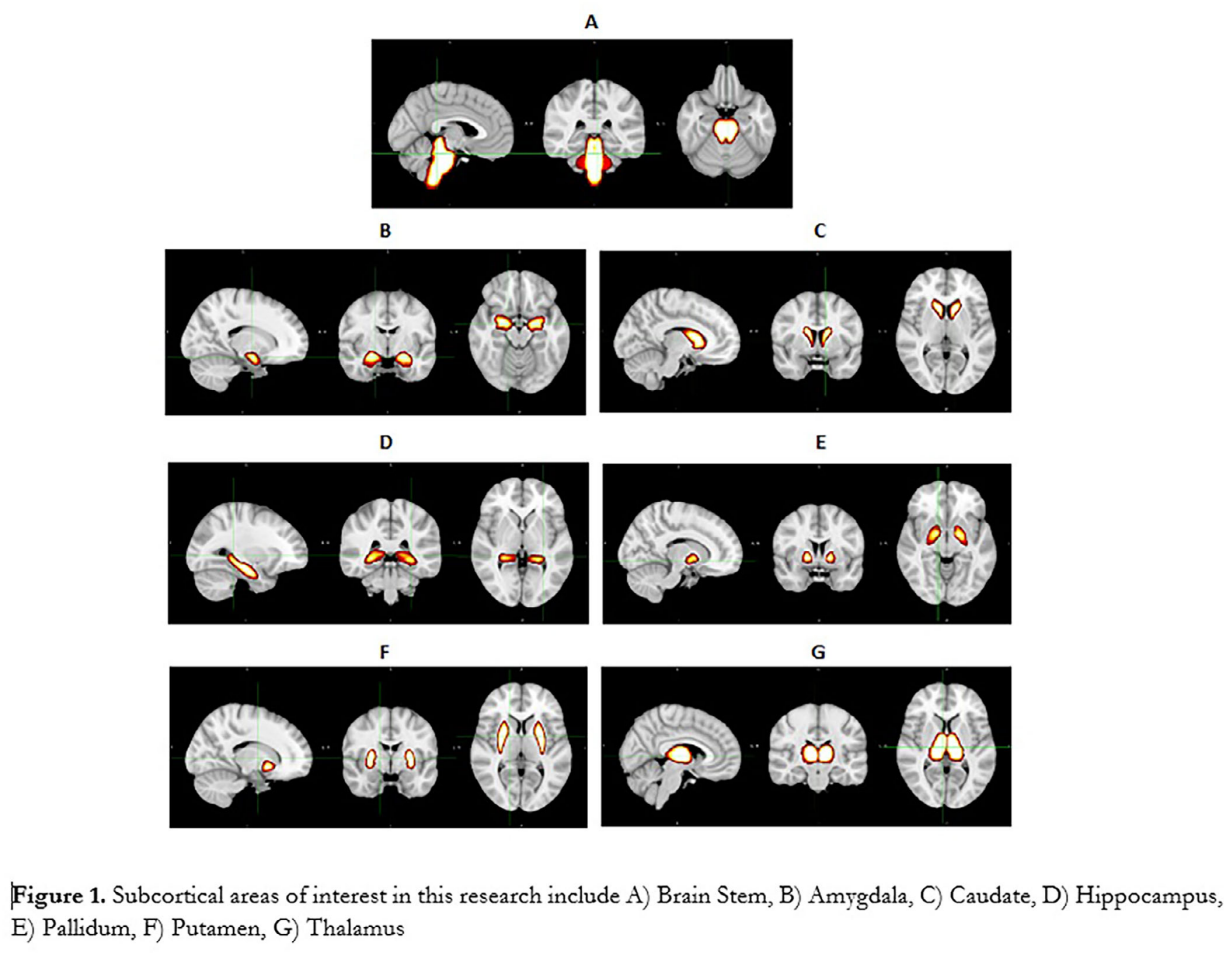# Distinct patterns of age‐related volume alterations in subcortical grey matter

**DOI:** 10.1002/alz70856_101472

**Published:** 2025-12-25

**Authors:** Fatemeh Tabassi Mofrad, John Gallacher

**Affiliations:** ^1^ University of Oxford, Oxford, United Kingdom; ^2^ University of Oxford, Oxford, Oxfordshire, United Kingdom

## Abstract

**Background:**

Age‐related cognitive decline has inevitable consequences for one's self‐sufficiency and life expectancy; yet, the associated brain structural changes have not been fully documented. Given the involvement of subcortical areas in deterioration of cognitive abilities, in this study we aimed to map the age‐related volumetric characteristics of grey matter in subcortical brain (Figure 1).

**Method:**

We used T1‐weighted structural brain scans data from 46,111 healthy participants; they were divided into the middle‐aged group (ages 44‐60) and the older group (ages 61‐83). A path analysis was performed to assess the relationship between the age and the volumes of subcortical areas. The goodness‐of‐fit of the model was evaluated using several fit indices, including the RMSEA, the CFI and the TLI. Based on modification indices, multicollinearities were adjusted and the model was re‐estimated to achieve a better fit.

**Result:**

According to our findings, age had significant positive direct effects resulting in increased volumes in the right and left Caudate, and Pallidum, and in the left Putamen; besides, age had significant negative direct effects and thus shrinkage in the volumes of the Brain Stem, the left Thalamus, the right and the left Amygdala, and Hippocampus in the older group.

**Conclusion:**

The analyses reveal that shrinkage of grey matter volume in subcortical areas is not the only reason behind age‐related changes in structural brain characteristics; increases in the volumes of the left and right Caudate and Pallidum and the left Putamen in the older group demonstrate age‐related brain inflammation due to immune system alterations, probably preceding pathogenesis of neurodegenerative diseases in some individuals.